# Optimizing the Fermentation Conditions of *Cudrania tricuspidata* Fruit Using *Bacillus amyloliquefaciens* for Anti-Inflammatory Activity and GC-MS-Based Volatile Component Characteristics

**DOI:** 10.1155/2023/5042416

**Published:** 2023-10-18

**Authors:** Si Young Ha, Ji Young Jung, Hyeon Cheol Kim, Jae-Kyung Yang

**Affiliations:** Department of Environmental Materials Science, Institute of Agriculture and Life Science, Gyeongsang National University, Jinju 52828, Republic of Korea

## Abstract

The aim of this study is to optimize the performance conditions used for maximum anti-inflammatory activity and to clarify *in vitro*anti-inflammatory properties of fermented *C. tricuspidata* fruit. Based on the single-factor experiment and Box–Behnken design, the optimized fermentation conditions of *C. tricuspidata* fruit for maximum anti-inflammatory activity were 3.8 d fermentation period, 8.4% (v/w) inoculation concentration, and 29.2°C fermentation temperature. Under optimal conditions, anti-inflammatory activity-based nitric oxide of fermented *C. tricuspidata* fruit reached 93.9%. Moreover, this study provides a theoretical basis and experimental data containing *β*-hexosaminidase and reactive oxygen species for the medical use and industrialization of *C. tricuspidata* fruit fermentation. Interestingly, the results of GC-MS analysis confirmed that fermented *C. tricuspidata* fruits detect volatile components different from unfermented *C. tricuspidata* fruits. We suggested that this volatile component may have been involved in the anti-inflammatory reaction, but scientific verification of this is needed later. Therefore, an in-depth study of volatile components detected from fermented *C. tricuspidata* fruits will need to be conducted later.

## 1. Introduction

*Cudrania tricuspidata* (*C. tricuspidata*), a small deciduous tree of the *Moraceae* family, is widely distributed throughout East Asia and is commonly referred to as cudrang or mandarin melon berry [1]. Previous studies have reported that *C. tricuspidata* is one of the common folk remedies for preventing cancer over the past few decades [2]. *C. tricuspidata* fruit has several health benefits [3]; however, most studies on its leaves and roots have been conducted because the raw fruits have unique flavors that are difficult for consumers to accept [4–6]. In addition, the fruits contain relatively fewer polyphenols and flavonoids than other parts of the plant, such as leaves and roots [7]. *C. tricuspidata* fruit has an extremely short shelf life and high moisture content, making it vulnerable to mechanical damage and microbial injury. This is similar to the characteristics of other berries [8, 9]. Therefore, there is an increasing trend in the postprocessing of *C. tricuspidata* fruits into vinegar, capsules, beverages, and powders [10, 11]. Previous studies have reported that the addition of appropriate processes to *C. tricuspidata* fruits significantly improves their functional properties and health characteristics [12, 13].

*C. tricuspidata* fruit has been used in several studies for its various medicinal properties. *C. tricuspidata* extracts have been reported to have therapeutic effects on various diseases, including cancer [14]. Park et al. [15] reported that flavonoids isolated from chloroform extracts from *C. tricuspidata* showed a significant inhibitory effect on NO production and iNOS expression in RAW264.7 cells. In addition, they have been demonstrated to have various medicinal effects, including prostaglandin E2 production in macrophages [16].

Chronic inflammatory diseases are the leading cause of mortality worldwide [17]. Recently, considerable attention has been focused on exploring the potential role of natural substances in the prevention and treatment of chronic inflammatory diseases [18–20]. Natural fermentation products are popular worldwide [21]. These products are rich sources of natural compounds, such as probiotics, with known biological properties [22]. Therefore, these fermented products have significant potential as natural substances for the treatment of inflammatory diseases. Fermentation has been used for a long time to preserve food. However, it has recently garnered considerable attention owing to an increase in the nutritional value of food and the production of health-promoting ingredients [23]. Microorganisms used in fermentation metabolize fermentable carbohydrates to produce bioactive ingredients [24, 25]. The active ingredients produced by fermentation can protect against diseases, including allergies [26]. Kim et al. [27] reported that fermented *Zizyphus jujuba* played a protective role against inflammation induced by nitric oxide (NO) and that the fermentation process improved anti-inflammatory activity. Seo et al. [28] reported that the total polyphenol content improved by 47% by organic acid fermentation of *C. tricuspidata* fruits. This was regarded as immune activity in fermented *C. tricuspidata* fruits, as the levels of interleukin (IL)-2 and IL-4 secretion increased. Johnson et al. [29] reported that fermentation improves the antioxidant capacity and increases the polyphenol content, causing anti-inflammatory activity in berries.

Although probiotic microorganisms guarantee the fermentation process, they can be destroyed during heating to produce fermented products without living microorganisms [30]. However, the fermentation process before pasteurization enriches fermented foods by releasing active metabolites from existing plant nutrients or through the fermentation process [31]. Therefore, it is important to determine the optimal fermentation conditions to preserve microorganisms in certain foods and enhance their medicinal effects. In fact, the findings of Yusuf et al. [32] indicate that the optimal fermentation conditions of total mixed rations with cottonseed meal or rapeseed meal enhance the nutritional value, thereby making them viable and usable feedstuffs for potential use in livestock industries.

The first goal of this study was to select *Lactobacillus* suitable for the fermentation of *C. tricuspidata* fruits based on OD600, pH, and solubility. Finally, the selected *Lactobacillus* strains were used to optimize the fermentation conditions to maximize the anti-inflammatory effect, based on the NO inhibition rate of the fermented *C. tricuspidata* fruit. This study is expected to provide a fermentation technology to increase the anti-inflammatory effects of *C. tricuspidata* fruits and provide evidence of their efficacy.

## 2. Materials and Methods

### 2.1. Materials

*C. tricuspidata* fruits were purchased from Dada Farm (Andong City, Gyeongsangbuk-do, South Korea). Fresh, high-quality *C. tricuspidata* fruits (South Korea National Agricultural Products Quality Management Agency No. 23 Certification Authority; Certification number: No. 16303207) were selected for this study. *Lactobacillus* were obtained from the Korean Collection for Type Cultures (South Korea).

### 2.2. Fermentation of *C. tricuspidata* Fruit Using *Lactobacillus*

The mature *C. tricuspidata* fruit was cleaned and fermented in a 1–5 d fermentation period, 2–10% inoculation concentration, and 24–35°C temperature. We used whole fruits during fermentation. It is understood that there is no universal strain or species that can provide a complete range of benefits. Therefore, the current work focused on screening anti-inflammation activities of seven available *Lactobacillus* strains: *Bacillus coagulans*, *Bacillus subtilis* subsp. *inaquosorum*, *Bacillus amyloliquefaciens*, *Enterococcus faecalis*, *Enterococcus faecium*, *Lactobacillus acidophilus*, and *Lactobacillus delbrueckii* subsp. *bulgaricus* (Table 1). After fermentation, the juice produced during the fermentation process was freeze-dried for 48 h to remove moisture. The dried juice was stored at −80°C before use to maintain that *Lactobacillus* no longer grows and completely dissolved in a culture medium containing dimethyl sulfoxide (DMSO) before use in the cell experiments.

### 2.3. Absorbance (OD600), pH, and Total Soluble Solids of Juice Obtained from Fermented *C. tricuspidata* Fruit

*Lactobacillus* were obtained by determining the 600 nm (OD600 nm) optical density [33], pH (AOAC 942.15), and total soluble solids (°Brix) (AOAC 932.12) [34].

### 2.4. NO Expression Analysis

The nitrite accumulated in the fermented *C. tricuspidata* was measured as an indicator of nitric oxide production according to the Griess reaction described by Green et al. [35] with slight modification. In detail, 100 *μ*L of each sample was mixed with 50 *μ*L of 1% sulfanilamide (in 5% phosphoric acid) and 50 *μ*L of 0.1% naphthalenediamine dihydrochloride and then incubated at room temperature for 10 min. The absorbance of the soluble purple products was measured at 550 nm using an ELISA plate reader (SpectraMax 190, Molecular Devices, LLC). For the standard curve, absorbance at 550 nm was measured with a NaNO_2_ serial dilution standard curve from 0 to 100 *μ*M, and nitrite production was determined.

### 2.5. Cell Toxicity Test

RAW264.7 and RBL-2H3 cell toxicity tests were analyzed using the 3-(4,5-dimethyl-2-thiazolyl)-2,5-diphenyltetrazolium bromide (MTT) assay. The RBL-2H3 and RAW264.7 cell lines were obtained from the Korean Cell Line Bank (Seoul, South Korea). Sequential twofold dilutions were performed to produce four concentrations (250, 500, 750, and 1000 ppm). The final dilution used for treating the cells contained not more than 1% of the dimethyl sulfoxide. The culture was then continued for 48 hours [36]. At the end of the exposure time, cell growth was analyzed using the MTT assay. The absorbance was measured at 540 nm using an absorbance microplate reader (SpectraMax 190; Molecular Devices LLC, CA, USA) [37].

### 2.6. Box–Behnken Experimental Design of Response Surface Methodology for Optimization of Variables

The Box–Behnken design was used to determine the optimum levels of the most significant variables (fermentation period, *X*_1_; inoculation concentration, *X*_2_; and fermentation temperature, *X*_3_) and study their interactions. The predicted response (*Y*) was the inhibition rate of NO (%) determined by measuring the survival rate of CCD-986sk cells. Each effective variable in the design was studied at three different levels (coded as −1, 0, and +1). A total of 17 experiments were conducted, and the entire experimental design considered three center points. The fitness of the second-order polynomial model was expressed through the regression coefficient *R*^2^, and a detailed analysis of variance was conducted at the coded level to determine the effects of individual variables. Statistical Analysis System (SAS) software (version 11.0; SAS Institute Inc., Cary, NC, USA) was used for regression and graphical analyses of the experimental data.

### 2.7. The *β*-Hexosaminidase Assay

RBL-2H3 cells were dispensed in 24-well plates at a concentration of 5 × 10^5^ cells/well. The medium was contained with penicillin, streptomycin, and anti-DNP IgE. The cells were incubated overnight at 37°C in a 5% CO_2_ incubator. The cells were washed twice with piperazine-N, N-bis-(2-ethanesulfonic acid) buffer. This medium was cultured at 37°C for 10 min in a PIPES buffer. Cells were treated with optimized fermentation juice (250–1000 ppm) or 20 *µ*M quercetin (positive control). After that, the cells were incubated at 37°C for 20 min, and the cells were activated by treating them with 25 ng/mL of antigen DNP-BSA for 30 min at 37°C. The supernatant was cultured with a 1 mM substrate (p-nitrophenyl-N-acetyl-*β*-D-glucosaminide) at 37°C for 1 h. The reaction was stopped by addition of 2 ml of 200 mM glycine NaOH buffer pH 10.6. The absorbance of the reaction solution was measured at 405 nm using a microplate reader (SpectraMax 190 Molecular Devices, LLC) [38].

### 2.8. ROS Analysis

Intracellular ROS levels were measured using DCFH-DA according to a previously described method [39]. RAW264.7 cells were seeded in 96-well plates at a density of 5 × 10^5^ cells per well and incubated for 24 hours. The cells were then treated with different concentrations of 250–1000 ppm fermented *C. tricuspidata*, or the 20 *µ*M quercetin (positive control) for 72 hours. After treatment, DCFH-DA was added to the cells and incubated for 30 minutes. The fluorescence intensities were measured using a microplate reader (SpectraMax 190, Molecular Devices LLC) at an excitation wavelength of 504 nm and an emission wavelength of 524 nm.

### 2.9. Gas Chromatography-Mass Spectrometry (GC-MS) Analysis

Volatile compounds were extracted from *C. tricuspidata* fruits using headspace solid-phase microextraction (HS-SPME) and analyzed via gas chromatography-mass spectrometry (GC-MS). The GC-MS analysis was performed using an Agilent 6890N GC DB-5 MS fused silica capillary column (30 mm × 0.25 mm i.d., film thickness 0.25 *μ*m). The GC-MS was equipped with an electron ionization system with an ionization energy of 70 eV, and the carrier gas used was helium at a constant flow rate of 1 mL/min. The injector and MS transfer line temperatures were set at 280°C and 250°C, respectively. The initial oven temperature was 50°C and was maintained for 2 min, then increased to 250°C at a rate of 10°C/min, and held at 250°C for 10 min. Diluted samples (1/100, v/v, in methanol) of 1.0 *μ*L were injected manually in the splitless mode. The relative percentages of components were expressed as percentages by peak area normalization. The identification of components was based on GC retention time on a DB-5 capillary column relative to computer matching of mass spectra using NIST libraries for the GC-MS system (software: NIST MS Search Program version 2.3).

### 2.10. Statistical Analysis

Data are presented as mean ± standard deviation (*n* = 3). Statistical analyses of the results were performed at a 5% significance level. Differences between the means of individual groups were assessed using Student's *t*-test (language R, R Development Core Team 2020, Vienna, Austria) and Duncan's multiple range test (SAS, SAS Institute, Inc., North Carolina, USA).

## 3. Results

### 3.1. Effect of the Strains on the Fermentation of *C. tricuspidata*

**Figure 1:**
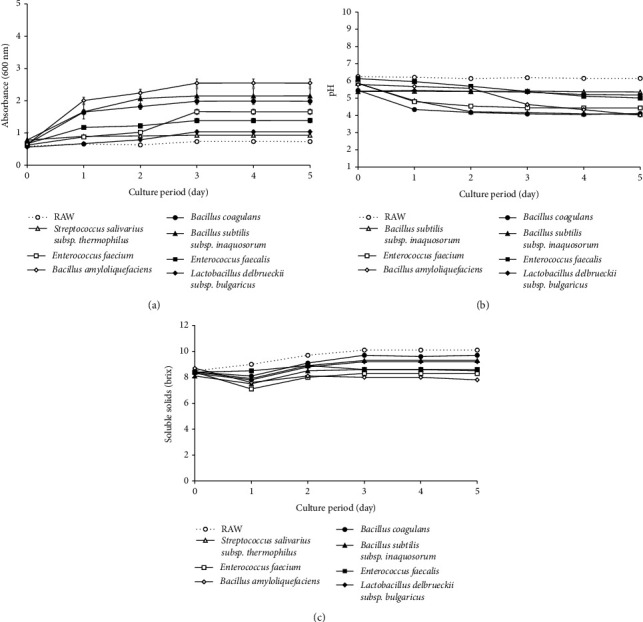
Effect of various *Lactobacillus* on the (a) viability (absorbance 600 nm), (b) pH, and (c) soluble solids in *C. tricuspidata* fruits.

**Figure 2:**
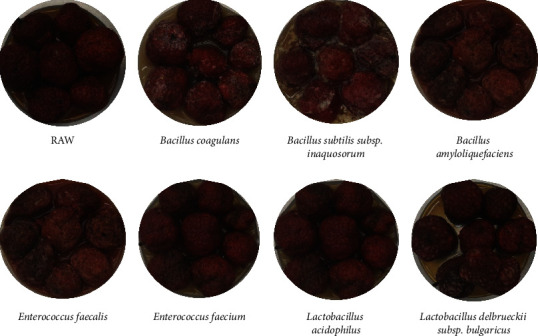
Visual variation of *C. tricuspidata* fruits fermented with various *Lactobacillus*.

The *Lactobacillus* density for fermentation was evaluated by measuring OD600 (Figure 1(a)). The optical density (OD) using a spectrophotometer of increasing microbial mass of various *Lactobacillus* during incubation at 37°C was measured in the presence of *C. tricuspidata* at different culture periods [40]. Fermentation with *B. amyloliquefaciens* (OD600, nm = 2.547) exhibited higher OD values than raw fermentation or fermentation with other *Lactobacillus* (OD600, nm = 0.931–2.189) using only *C. tricuspidata* (OD600, nm = 0.734). This indicates that *B. amyloliquefaciens* is suitable for fermentation with *C. tricuspidata*. A photograph of *C. tricuspidata* fermented using *B. amyloliquefaciens* also showed the occurrence of fermentation (Figure 2). Visual changes in *C. tricuspidata* during fermentation for 5 days are shown in Figure 2.

This study demonstrated the significant effects of *Lactobacillus* growth on the physicochemical parameters (pH and soluble solids; Figures 1(b) and 1(c)) of fermented *C. tricuspidata*, including nonvaccination controls. The pH decreased by approximately 1-2 during lactobacillus treatment, probably because of the conversion of sugars to organic acids (e.g., malic and lactic acids) [41], and the initial pH value also decreased. *Lactobacillus* culture medium decreases the pH by producing acid-based substances. It has been reported that this increases the acidity of the medium, creating an environment unfavorable to pathogens [42]. *C. tricuspidata* juice fermented using *Lactobacillus* exhibited a significant difference from the nonvaccinated control (raw) in the graph as a result of the pH comparison according to the incubation period.

Figure 1(c) shows the effect of *Lactobacillus* on the total soluble solid content of *C. tricuspidata*. The soluble solids content (°Brix) of *C. tricuspidata* containing *Lactobacillus* was lower than that of *C. tricuspidata* without *Lactobacillus*. The main sources of carbon and energy for *Lactobacillus* are glucose and fructose, respectively. Braga and Conti-Silva [43] reported a tendency of total soluble solids, similar to our results in papaya connectors with added *Lactobacillus*. Therefore, during fermentation, the solid content of the fruit tended to decrease. These observations confirm the characteristics of fruit-based fermentation. In conclusion, *B. amyloliquefaciens* exhibited an OD600 > 2.0 after one day of incubation, indicating that they could survive better in *C. tricuspidata* than in other media.

### 3.2. Effects of Fermented *C. tricuspidata* with Various *Lactobacillus* on NO in Macrophages

**Figure 3:**
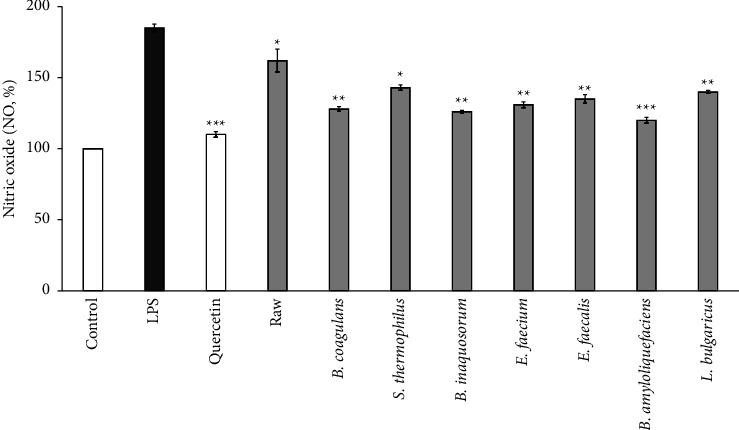
The effects of fermented *C. tricuspidata* on the NO of RAW264.7 cells. The data are represented as the standard deviation of three independent experiments. ^*∗*^indicates a significant difference (*p* < 0.05), ^*∗∗*^indicates a significant difference (*p* < 0.01), and ^*∗∗∗*^indicates a significant difference (*p* < 0.001) compared with the LPS. Control: nontreated lipopolysaccharide; LPS: treated lipopolysaccharide; raw: treated lipopolysaccharide and nonfermented *C. tricuspidata*.

NO is an inflammation-inducing mediator linked to various diseases [44]. Overproduction of NO activates inflammation-related tissue damage and infection [45]. In this study, NO inhibition was measured after treatment with *C. tricuspidata* fermented with various *Lactobacillus* strains. A noticeable increase in NO metabolites (1.8-fold) was observed in LPS-treated RAW264.7 (Figure 3) compared to the untreated control group (see Control). However, fermented *C. tricuspidata* significantly (*p* < 0.01) reduced the expression of NO metabolites in the cell lines. Notably, a higher NO inhibition rate was observed in *C. tricuspidata* after fermentation than before fermentation. Fermented fruits exhibit high bioactivities, such as antioxidant and anti-inflammatory properties [46, 47]. Previous studies have indicated that the anti-inflammatory active ingredients of *Trapa japonica* fruit extract can be supplemented by fermentation with two microorganisms, *Bacillus subtilis* and *Bacillus methylotrophicus* [48]. These results follow the same trend as that observed in our study. In particular, the fruit juice exhibited the lowest NO in *C. tricuspidata* fermented using *B. amyloliquefaciens*. *C. tricuspidata* fermented using *B. amyloliquefaciens* was a significant difference with the LPS treatment group (*p* < 0.001); however, it was similar to quercetin, a positive control. *B. amyloliquefaciens* belongs to the superfamily of bacteria belonging to the *Bacillaceae* family and genus *Bacillus* [49]. The previous study reported the investigated cytotoxic potential of *B. amyloliquefaciens* used in industrial production of enzyme products and discovered that none demonstrated any in vitro cytotoxic activity. [50].

The U.S. Food and Drug Administration (FDA) considers *B. amyloliquefaciens* generally safe for food and medical purposes [51]. Moreover, some strains of *B. amyloliquefaciens* also suppress the synthesis of toxic substances [52]. The presence of *B. amyloliquefaciens* in some products has been reported to improve product quality, increase hydrophilicity, and increase the amount of bioactive compounds [53]. We performed the subsequent analyses using *B. amyloliquefaciens*, which exhibited the lowest NO levels.

### 3.3. Cytotoxicity of the Fermentation Juice Obtained from *B. amyloliquefaciens* Fruits in RAW264.7 and RBL-2H3 Cells

**Figure 4:**
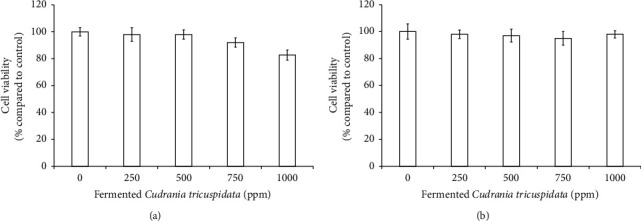
The effects of fermented *C. tricuspidata* fruits on the cell viability of RBL-2H3 (a) and RAW264.7 (b), respectively. The data are represented as the standard deviation of three independent experiments. ^*∗*^indicates a significant difference (*p* < 0.05) compared with the untreated group. However, there was no significance in these data.

We evaluated the cytotoxicity of the fermentation juice obtained from fermented *C. tricuspidata* fruit with *B. amyloliquefaciens* in RAW264.7 and RBL-2H3 cells. As shown in Figure 4, 1000 ppm juice induced nonsignificant cytotoxicity in RBL-2H3 (82.7%, *p* < 0.05). Moreover, the juice had no effect on RAW264.7 cells, even at a concentration of 1000 ppm. Therefore, in subsequent experiments, both RAW264.7 and RBL-2H3 cells were treated with nontoxic 1000 ppm concentrations of the juice.

### 3.4. Experimental Design

As little information is available on NO control in fermented *C. tricuspidata*, a fractional factorial 17 design was initially designed to investigate the influence of the fermentation period, *Lactobacillus* inoculation concentration, and fermentation temperature. The experimental design, factors, levels (coded and decoded), and responses are summarized in Table 2.

**Figure 5:**
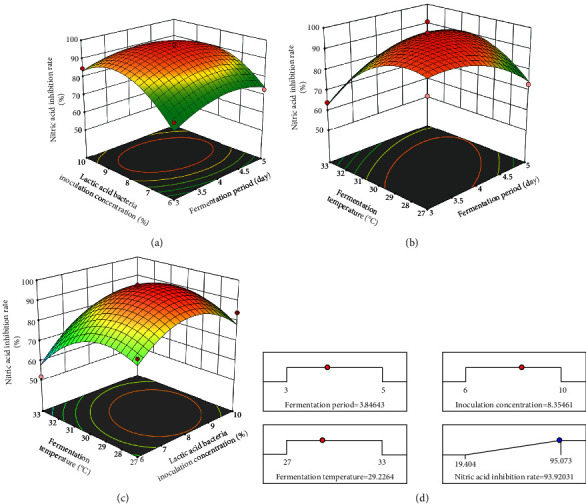
Regression analysis of the Box‐Behnken design experiments 3D plot. (a) Response surface graphs for nitric oxide inhibition rate as a function of fermentation period and inoculation concentration; (b) response surface graphs for nitric oxide inhibition rate as a function of fermentation period and fermentation temperature; (c) response surface graphs for nitric oxide inhibition rate as a function of inoculation concentration and fermentation temperature; (d) optimal fermentation conditions of *C. tricuspidata* and maximum nitric oxide inhibition rate.

The NO inhibition rate ranged from 19% to 85% of fermented *C. tricuspidata*. The highest inhibition rate (93.92%) was obtained when *C. tricuspidata* fermentation conditions were fermentation period of 3.8 days, inoculation concentration 8.4%, and fermentation temperature 29.2°C (Figure 5). The quadratic effects of the inoculation concentration had the greatest effect on the NO inhibition rate, as summarized in Table 3 (regression coefficient: 2.79). Therefore, the NO inhibition rate changed significantly with the controlled inoculation concentration (*p* < 0.05). This model was adopted as a model of the quadratic form, not as a linear one. Therefore, lack of fit can be significant, which can clarify the reliability of the model [54, 55].

The following equation expresses the statistical models for the NO inhibition rate:

(1) $$\begin{array}{c} \text{NO inhibition rate},\%=-42.10925-1.72628\times A+1.83467\times B+2.77921\times C-0.052500\times \text{AB}+0.159250\times \text{AC}+0.336670\times \text{BC}-0.323575\times A^{2}-0.156581\times B^{2}-0.062842\times C^{2}, \end{array}$$

where A is the fermentation period (days), B is the *Lactobacillus* inoculation concentration (%), and C is the fermentation temperature (°C).

We show the reliability test results for the measured values and predicted values in Figure S1 (Supplementary data). This result shows that the measured and predicted values are very similar.

### 3.5. Effects of Fermented *C. tricuspidata* on the *β*-Hexosaminidase Activity in DNP-BSA-Stimulated RBL-2H3 Cells

**Figure 6:**
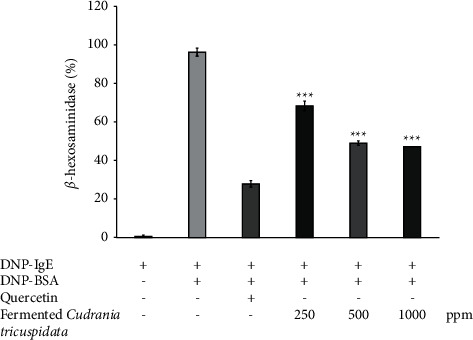
The effects of fermented *C. tricuspidata* on *β*-hexosaminidase induced by DNP-BSA from RBL-2H3 cells. The data are represented as the standard deviation of three independent experiments. ^*∗∗∗*^*p* value <0.001 compared with DNP-IgE + DNP-BSA.

We performed *β*-hexosaminidase assay to prove the anti-inflammatory effect of *C. tricuspidata* fermented under optimized conditions. We examined the effects of fermented *C. tricuspidata* on the DNP-BSA-induced release of *β*-hexosaminidase from RBL-2H3 cells [56]. RBL-2H3 cells were treated with fermented *C. tricuspidata* and quercetin as a positive control to evaluate their inhibitory effects on the release of *β*-hexosaminidase. All test samples significantly (*p* < 0.001) inhibited the release of *β*-hexosaminidase from antigen-stimulated RBL-2H3 cells (Figure 6).

Among them, fermented *C. tricuspidata* exhibited excellent inhibitory effects on *β*-hexosaminidase activity at concentrations ranging from 500 to 1000 ppm. These results demonstrated that fermented *C. tricuspidata* is a potent anti-inflammatory drug.

### 3.6. Effects of Fermented *C. tricuspidata* on ROS Level in LPS-Stimulated RAW264.7

**Figure 7:**
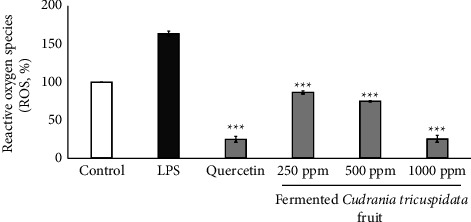
The effects of fermented *C. tricuspidata* on ROS induced by LPS from RAW264.7 cells. The data are represented as the standard deviation of three independent experiments. ^*∗∗∗*^*p* value <0.001 compared with LPS.

The effects of fermented *C. tricuspidata* on intracellular free radical production in LPS-stimulated RAW264.7 cells were analyzed. The level of ROS was similar to that of the positive control quercetin (Figure 7). LPS stimulation increased ROS production, which was significantly decreased upon treatment with different concentrations of fermented *C. tricuspidata*. These results suggest that fermented *C. tricuspidata*, which are considered to be involved in anti-inflammatory events, may stimulate the secretion of ROS.

### 3.7. Volatile Compounds of Fermented *C. tricuspidata*

According to the results of this paper, the fermented *C. tricuspidata* fruits contained various components including esters, alcohols, acids, ketones, volatile phenol, organic matter, and miscellaneous compounds. The major components were diethyl phthalate (27.85%), aristolone (11.16%), furfuryl alcohol (8.69%), and scyllitol (8.16%), as presented in Table 4, Figure S2 (Supplementary data), and Figure S3 (Supplementary data). To the best of our knowledge, the composition of fermented *C. tricuspidata* fruit has not been previously reported. Therefore, our findings represent the first report on the composition of fermented *C. tricuspidata* fruit.

## 4. Discussion

Anti-inflammatory response in the human body is crucially important for several reasons. Inflammation is generally a defense mechanism of the immune system in response to external stimuli such as injury, infection, irritants, and toxins However, if chronic inflammation occurs or the immune system continuously triggers inflammation, it can be detrimental to health. Chronic inflammation is associated with many diseases, such as cardiovascular disease, diabetes, cancer, atopic dermatitis, and rheumatoid arthritis. Thus, suppressing inflammation through anti-inflammatory drugs or food components is beneficial for overall health.

The results of this study demonstrated that fermented *C. tricuspidata* also has the ability to inhibit the production of NO, as measured in the Griess assay. Ryu et al. [57] reported that *Artemisia iwayomogi*, *Machilus thunbergii*, *Populus davidiana,* and *Populus maximowiczii* exhibited potent NO inhibition above 70%. Jabit et al. [58] reported high selectivity indices for NO inhibition in the stems of *Garcinia bancana* (stem) and *Garcinia malaccensis* (stem). Most studies have reported that plant leaves or stems effectively inhibit NO; however, reports on inhibition owing to fruits are insufficient. In this study, an NO inhibitory activity of 90% or more could be reached by fermenting the fruits. These results are expected to provide potent anti-inflammatory agents, which could pave the way for the discovery of novel clinical candidates.

*β*-Hexosaminidase is also considered a degranulation marker of mast cells [59]. Overproduction of free radicals during inflammatory processes is involved in signal transduction and NF-*κ*B activation. In this study, we suggest that fermented *C. tricuspidata*, which is considered to be involved in anti-inflammatory events, may stimulate the secretion of *β*-hexosaminidase or ROS.

In previous paper, short-chain fatty acids, including butanoic acid, 3-methyl butanoic acid, 2-methyl propanoic acid, pentanoic acid, and hexanoic acid, are characterized by unpleasant, acidic, parmesan cheese, sour fruit, apple peel, rancid, sharp, or pungent aromas [60]. 3-Methylbutanoic acid has pungent, cheesy, sweaty, or old sock sensory characteristics, but its esters have a fruity character. In our study, these same components were detected in both fermented and nonfermented *C. tricuspidata* fruits. Interestingly, in fermented *C. tricuspidata* fruits, all acids except 3-methylbutanoic acid were reduced, while all ketones except Lanost-8-en-3-one were newly detected. Furfuryl alcohol, which was detected in fermented *C. tricuspidata* fruits, belongs to the furan compound family. Previous studies have demonstrated that furan compounds can be a source of novel tyrosinase inhibitors and can be developed as bioinsecticides and antimicrobials [61]. Furfuryl alcohol, a sugar degradation product, inhibits aldehyde oxidation [62]. Furanones, which are formed from the Maillard reactions between carbohydrates and proteins, have antioxidant activity [63, 64]. Furthermore, some furanones are found in fruits, and their antioxidant and anti-inflammatory activities have been reported [65]. 2-Methoxy-4-vinylphenol, which is found in fermented *C. tricuspidata* fruits, possess antioxidant activity as well as anti-inflammatory activity [66]. Therefore, we assumed that the volatile substances found in fermented *C. tricuspidata* fruits affected anti-inflammatory activity.

## 5. Conclusion

To the best of our knowledge, this is the first study to determine the optimal fermentation conditions under which *C. tricuspidata* fruits maximally inhibit NO production. *B. amyloliquefaciens* was used in the optimal fermentation process of *C. tricuspidata* to maximize NO inhibition. This strain was confirmed to exhibit the highest NO inhibition rate among the seven *Lactobacillus* species. The obtained results verified that fermented *C. tricuspidata* under optimal conditions significantly suppressed the production of proinflammatory markers *β*-hexosaminidase induced by DNP-BSA from RBL-2H3 cells. Therefore, we demonstrated that fermented *C. tricuspidata* could inhibit inflammation from harmful factors such as NO or *β*-hexosaminidase. The results of GC-MS analysis were interesting, as they confirmed the presence of volatile components in fermented *C. tricuspidata* fruits that were not detected in unfermented *C. tricuspidata* fruits. We suggested that these volatile components may be involved in the anti-inflammatory reaction, but scientific verification is required in future studies.

## Figures and Tables

**Table 1:** Lactobacillus used for the fermentation of *C. tricuspidata* fruits.

No.	Strain	KACC no.	Media	Temperature (°C)
1	*Bacillus coagulans*	11248	MRS broth	37
2	*Bacillus subtilis subsp. inaquosorum*	19623	Trypticase soy broth	28
3	*Bacillus amyloliquefaciens*	17107	Trypticase soy broth	28
4	*Enterococcus faecalis*	13807	Trypticase soy broth	28
5	*Enterococcus faecium*	15684	Trypticase soy broth	28
6	*Lactobacillus acidophilus*	12419	MRS broth	37
7	*Lactobacillus delbrueckii subsp. bulgaricus*	12420	MRS broth	37

**Table 2:** Results of three-factor Box–Behnken experimental design.

Std.	Run	Fermentation period (days)	Inoculation concentration (%)	Fermentation temperature (°C)	Nitric oxide inhibition rate (%, actual)	Nitric oxide inhibition rate (%, predicted)
8	1	5	8	33	83.89	82.16
11	2	4	6	33	69.40	60.88
7	3	3	8	33	89.77	81.12
13	4	4	8	30	75.01	75.51
9	5	4	6	27	54.35	50.89
16	6	4	8	30	75.07	75.51
10	7	4	10	27	82.96	83.72
17	8	4	8	30	70.35	75.51
5	9	3	8	27	63.33	68.04
3	10	3	10	30	93.89	93.07
12	11	4	10	33	94.68	101.80
14	12	4	8	30	75.57	75.51
4	13	5	10	30	92.99	92.94
1	14	3	6	30	54.75	55.98
15	15	4	8	30	74.91	75.51
2	16	5	6	30	54.71	56.27
6	17	5	8	27	64.38	67.17

**Table 3:** ANOVA results of the fit model from Box–Behnken design.

Source	Sum of squares	*df*	Mean square	*F*-value	*P* value	
Model	8077.33	9	0.6664	6.80	0.0097	Significant
A-period	3972.08	1	0.0145	0.1483	0.7116	
B-concentration	5.53	1	0.5356	5.47	0.0520	
C-temp	516.01	1	0.5197	5.30	0.0548	
AB	184.96	1	0.0441	0.4500	0.5238	
AC	428.49	1	0.9130	9.32	0.0185	
BC	25.96	1	0.1632	1.67	0.2378	
A^2^	2688.25	1	0.4408	4.50	0.0716	
B^2^	157.72	1	1.65	16.86	0.0045	
C^2^	1.14	1	1.35	13.74	0.0076	
Residual	559.42	7	0.0980			
Lack of fit	547.08	3	0.2070	12.73	0.0163	Significant
Pure error	12.34	4	0.0163			
Cor total	8636.76	16				

**Table 4:** Volatile compounds identified in fermented (or not) *C. tricuspidata.*

No.	Component	Peak area (%)	
		Nonfermented	Fermented
*Esters*			
1	2-Methyl-2-methylpropyl propanoate	—	2.81
2	2-Octyl benzoate	—	1.37
3	Ethyl hexadecanoate	0.88	1.33
4	Diethyl phthalate	33.10	27.85
5	Methyl linoleate	0.46	0.31
6	Butyl citrate	5.12	3.30

*Alcohols*			
7	1-Hexanol	—	5.16
8	2,3,4-Trimethyl-3-pentanol	—	1.11
9	Furfuryl alcohol	—	8.69
10	Scyllitol	15.01	8.16
11	p-Hydroxybenzyl alcohol	4.05	1.31
12	Vitamin E	1.11	0.52
13	Campesterol	1.06	1.19

*Acids*			
14	3-Methyl-butanoic acid	—	2.17
15	Boric acid	3.78	1.55
16	Syringic acid	1.16	0.19
17	Palmitic acid	6.33	2.11
18	(Z,Z)-9,12-Octadecadienoic acid	1.07	0.13
19	Stearic acid	2.05	1.95
20	2-Methyl-propanoic acid	0.73	—
21	Butanoic acid	1.23	—
22	3-Methyl-butanoic acid	0.54	0.47
23	Pentanoic acid	0.37	0.33
24	Hexanoic acid	0.64	0.39

*Ketones*			
25	2,3-Butanedione	—	2.22
26	3-Hydroxy-2-butanone	—	0.65
27	Dihydro-5-pentyl-2(3H)-furanone	—	1.38
28	Lanost-8-en-3-one	3.02	2.85

*Volatile phenol*			
29	2-Methyl-4-vinylphenol	—	4.15
30	Aristolone	12.11	11.16

*Organic matter*			
31	Lanol	1.13	0.85

*Miscellaneous*			
32	Quinindoline	5.05	4.34

*Note.* “—” denotes not detected.

## Data Availability

The data used to support the findings of this study are available from the corresponding author upon request.

## Supplementary Materials

Supplementary Materials Figure S1 shows the plot of actual vs. predicted response for nitric oxide inhibition rate. Figure S2 and Figure S3 show the total ion chromatograph of nonfermented and fermented *C. tricuspidata*, respectively.

## References

[1] Xin L. T., Yue S. J., Fan Y. C. (2017). *Cudrania tricuspidata*: an updated review on ethnomedicine, phytochemistry and pharmacology. *RSC Advances*.

[2] Li X., Yao Z., Jiang X. (2020). Bioactive compounds from *Cudrania tricuspidata*: a natural anticancer source. *Critical Reviews in Food Science and Nutrition*.

[3] Byun E. B., Song H. Y., Kim W. S. (2021). Protective effect of polysaccharides extracted from *Cudrania tricuspidata* fruit against cisplatin-induced cytotoxicity in macrophages and a mouse model. *International Journal of Molecular Sciences*.

[4] Jeong C. H., Choi G. N., Kim J. H. (2010). Protective effects of aqueous extract from *Cudrania tricuspidata* on oxidative stress-induced neurotoxicity. *Food Science and Biotechnology*.

[5] Cho S. S., Yang J. H., Seo K. H. (2019). *Cudrania tricuspidata* extract and its major constituents inhibit oxidative stress-induced liver injury. *Journal of Medicinal Food*.

[6] Kim Y. S., Lee Y., Kim J. (2012). Inhibitory activities of Cudrania tricuspidata leaves on pancreatic lipase in vitro and lipolysis in vivo. *Evidence-based Complementary and Alternative Medicine*.

[7] Lee S. B., Cosmas B., Park H. D. (2020). The antimutagenic and antioxidant activity of fermented milk supplemented with *Cudrania tricuspidata* powder. *Foods*.

[8] Kusumaningrum D., Lee S. H., Lee W. H., Mo C., Cho B. K. (2015). A review of technologies to prolong the shelf life of fresh tropical fruits in Southeast Asia. *Journal of Biosystems Engineering*.

[9] Pareek S., Benkeblia N., Janick J., Cao S., Yahia E. M. (2014). Postharvest physiology and technology of loquat (*Eriobotrya japonica* lindl.) fruit. *Journal of the Science of Food and Agriculture*.

[10] Jeong J. Y., Jo Y. H., Lee K. Y., Do S. G., Hwang B. Y., Lee M. K. (2014). Optimization of pancreatic lipase inhibition by *Cudrania tricuspidata* fruits using response surface methodology. *Bioorganic & Medicinal Chemistry Letters*.

[11] Ousaaid D., Mechchate H., Laaroussi H. (2021). Fruits vinegar: quality characteristics, phytochemistry, and functionality. *Molecules*.

[12] Bajpai V. K., Sharma A., Baek K. H. (2013). Antibacterial mode of action of *Cudrania tricuspidata* fruit essential oil, affecting membrane permeability and surface characteristics of food-borne pathogens. *Food Control*.

[13] Oh N. S., Lee J. Y., Joung J. Y. (2016). Microbiological characterization and functionality of set-type yogurt fermented with potential prebiotic substrates *Cudrania tricuspidata* and *Morus alba* L. leaf extracts. *Journal of Dairy Science*.

[14] Jiang X., Cao C., Sun W. (2019). Scandenolone from *Cudrania tricuspidata* fruit extract suppresses the viability of breast cancer cells (MCF-7) *in vitro* and *in vivo*. *Food and Chemical Toxicology*.

[15] Park K. H., Park Y. D., Han J. M. (2006). Anti-atherosclerotic and anti-inflammatory activities of catecholic xanthones and flavonoids isolated from *Cudrania tricuspidata*. *Bioorganic & Medicinal Chemistry Letters*.

[16] Yang G., Lee K., Lee M., Ham I., Choi H. Y. (2012). Inhibition of lipopolysaccharide-induced nitric oxide and prostaglandin E2 production by chloroform fraction of *Cudrania tricuspidata* in RAW 264.7 macrophages. *BMC Complementary and Alternative Medicine*.

[17] Alsayari A., Wahab S. (2021). Genus ziziphus for the treatment of chronic inflammatory diseases. *Saudi Journal of Biological Sciences*.

[18] Kim J. H., Kismali G., Gupta S. C. (2018). Natural Products for the Prevention and Treatment of Chronic Inflammatory Diseases: Integrating Traditional Medicine into Modern Chronic Diseases Care. *Evidence-Based Complementary and Alternative Medicine*.

[19] Jantan I., Haque M. A., Arshad L., Harikrishnan H., Septama A. W., Mohamed-Hussein Z. A. (2021). Dietary polyphenols suppress chronic inflammation by modulation of multiple inflammation-associated cell signaling pathways. *The Journal of Nutritional Biochemistry*.

[20] Barakat A. Z., Hamed A. R., Bassuiny R. I., Abdel-Aty A. M., Mohamed S. A. (2020). Date palm and saw palmetto seeds functional properties: antioxidant, anti-inflammatory and antimicrobial activities. *Journal of Food Measurement and Characterization*.

[21] Soni S., Dey G. (2014). Perspectives on global fermented foods. *British Food Journal*.

[22] Marco M. L., Sanders M. E., Gänzle M. (2021). The international scientific association for probiotics and prebiotics (ISAPP) consensus statement on fermented foods. *Nature Reviews Gastroenterology & Hepatology*.

[23] Melini F., Melini V., Luziatelli F., Ficca A. G., Ruzzi M. (2019). Health-promoting components in fermented foods: an up-to-date systematic review. *Nutrients*.

[24] Hugenholtz F., Mullaney J. A., Kleerebezem M., Smidt H., Rosendale D. I. (2013). Modulation of the microbial fermentation in the gut by fermentable carbohydrates. *Bioactive Carbohydrates and Dietary Fibre*.

[25] El-Shishtawy R. M., Mohamed S. A., Asiri A. M., Gomaa A., Ibrahim I. H., Al-Talhi H. A. (2015). Saccharification and hydrolytic enzyme production of alkali pre-treated wheat bran by *Trichoderma virens* under solid state fermentation. *BMC Biotechnology*.

[26] Cross M. L., Stevenson L. M., Gill H. S. (2001). Anti-allergy properties of fermented foods: an important immunoregulatory mechanism of lactic acid bacteria. *International Immunopharmacology*.

[27] Kim M. J., Jung J. E., Lee S., Cho E. J., Kim H. Y. (2021). Effects of the fermented *Zizyphus jujuba* in the amyloid *β*25-35-induced Alzheimer’s disease mouse model. *Nutrition Research and Practice*.

[28] Seo M. J., Kang B. W., Park J. U. (2013). Effect of fermented *Cudrania tricuspidata* fruit extracts on the generation of the cytokines in mouse spleen cells. *Journal of Life Sciences*.

[29] Ha J., Oh H., Oh N. S. (2020). Anti-inflammatory effect of a peptide derived from the synbiotics, fermented *Cudrania tricuspidata* with *Lactobacillus gasseri*, on inflammatory bowel disease. *Mediators of Inflammation*.

[30] Admassie M. A. (2018). A review on food fermentation and the biotechnology of lactic acid bacteria. *World Journal of Food Science and Technology*.

[31] Xiang H., Sun-Waterhouse D., Waterhouse G. I., Cui C., Ruan Z. (2019). Fermentation-enabled wellness foods: a fresh perspective. *Food Science and Human Wellness*.

[32] Yusuf H. A., Piao M., Ma T., Huo R., Tu Y. (2021). Enhancing the quality of total mixed ration containing cottonseed or rapeseed meal by optimization of fermentation conditions. *Fermentation*.

[33] Adebola O. O., Corcoran O., Morgan W. A. (2014). Synbiotics: the impact of potential prebiotics inulin, lactulose and lactobionic acid on the survival and growth of lactobacilli probiotics. *Journal of Functional Foods*.

[34] Aoac (1998). *Official Methods of Analysis*.

[35] Green L. C., Wagner D. A., Glogowski J., Skipper P. L., Wishnok J. S., Tannenbaum S. R. (1982). Analysis of nitrate, nitrite, and [15N] nitrate in biological fluids. *Analytical Biochemistry*.

[36] Panichayupakaranant P., Tewtrakul S., Yuenyongsawad S. (2010). Antibacterial, anti-inflammatory and anti-allergic activities of standardised pomegranate rind extract. *Food Chemistry*.

[37] Bahuguna A., Khan I., Bajpai V. K., Kang S. C. (2017). MTT assay to evaluate the cytotoxic potential of a drug. *Bangladesh Journal of Pharmacology*.

[38] Matsuda H., Tewtrakul S., Morikawa T., Yoshikawa M. (2004). Anti-allergic activity of stilbenes from Korean rhubarb (*Rheum undulatum* L.): structure requirements for inhibition of antigen-induced degranulation and their effects on the release of TNF-*α* and IL-4 in RBL-2H3 cells. *Bioorganic & Medicinal Chemistry*.

[39] Shin Y. J., Cho D. Y., Chung T. Y., Han S. B., Hyon J. Y., Wee W. R. (2011). Rapamycin reduces reactive oxygen species in cultured human corneal endothelial cells. *Current Eye Research*.

[40] Sher S., Hussain S. Z., Rehman A. (2020). Phenotypic and genomic analysis of multiple heavy metal–resistant Micrococcus luteus strain AS2 isolated from industrial waste water and its potential use in arsenic bioremediation. *Applied Microbiology and Biotechnology*.

[41] Valero-Cases E., Frutos M. J. (2017). Effect of inulin on the viability of *L. plantarum* during storage and *in vitro* digestion and on composition parameters of vegetable fermented juices. *Plant Foods for Human Nutrition*.

[42] Pereira A. L. F., Maciel T. C., Rodrigues S. (2011). Probiotic beverage from cashew apple juice fermented with *Lactobacillus casei*. *Food Research International*.

[43] Braga H. F., Conti-Silva A. C. (2015). Papaya nectar formulated with prebiotics: chemical characterization and sensory acceptability. *LWT--Food Science and Technology*.

[44] Batista A. C., Silva T. A., Chun J. H., Lara V. S. (2002). Nitric oxide synthesis and severity of human periodontal disease. *Oral Diseases*.

[45] Yongvanit P., Pinlaor S., Bartsch H. (2012). Oxidative and nitrative DNA damage: key events in Opisthorchiasis-induced carcinogenesis. *Parasitology International*.

[46] Ayed L., M’hir S., Hamdi M. (2020). Microbiological, biochemical, and functional aspects of fermented vegetable and fruit beverages. *Journal of Chemistry*.

[47] Choo K. Y., Kho C., Ong Y. Y. (2018). Fermentation of red dragon fruit (*Hylocereus polyrhizus*) for betalains concentration. *International Food Research Journal*.

[48] Nam G. H., Jo K. J., Park Y. S. (2019). Bacillus/trapa Japonica fruit extract ferment filtrate enhances human hair follicle dermal papilla cell proliferation via the Akt/ERK/GSK-3*β* signaling pathway. *BMC Complementary and Alternative Medicine*.

[49] Cihan A. C., Yildiz E. D., Sahin E., Mutlu O. (2018). Introduction of novel thermostable *α*-amylases from genus anoxybacillus and proposing to group the Bacillaceae related *α*-amylases under five individual GH13 subfamilies. *World Journal of Microbiology and Biotechnology*.

[50] Bhat A. R., Irorere V. U., Bartlett T. (2013). *Bacillus subtilis* natto: a non-toxic source of poly-*γ*-glutamic acid that could be used as a cryoprotectant for probiotic bacteria. *AMB Express*.

[51] WoldemariamYohannes K., Wan Z., Yu Q. (2020). Prebiotic, probiotic, antimicrobial, and functional food applications of *Bacillus amyloliquefaciens*. *Journal of Agricultural and Food Chemistry*.

[52] Hanif A., Zhang F., Li P. (2019). Fengycin produced by *Bacillus amyloliquefaciens* FZB42 inhibits *Fusarium graminearum* growth and mycotoxins biosynthesis. *Toxins*.

[53] Chen G., Fang Q., Liao Z. (2022). Detoxification of aflatoxin B1 by a potential probiotic *Bacillus amyloliquefaciens* WF2020. *Frontiers in Microbiology*.

[54] Zaïd T. A., Bensari L., Benmaza K., Chitour C. E., Canselier J. P., Pierlot C. (2007). Response surface methodology as an approach to the optimization of a dishwashing detergent. *Tenside Surfactants Detergents*.

[55] Rath S. S., Pany A., Jayasankar K. (2013). Statistical modeling studies of iron recovery from red mud using thermal plasma. *Plasma Science and Technology*.

[56] Sohn E. H., Jang S. A., Joo H. (2011). Anti-allergic and anti-inflammatory effects of butanol extract from *Arctium Lappa* L. *Clinical and Molecular Allergy*.

[57] Ryu J. H., Ahn H., Kim J. Y., Kim Y. K. (2003). Inhibitory activity of plant extracts on nitric oxide synthesis in LPS‐activated macrophages. *Phytotherapy Research*.

[58] Jabit M. L., Wahyuni F. S., Khalid R. (2009). Cytotoxic and nitric oxide inhibitory activities of methanol extracts of *Garcinia* species. *Pharmaceutical Biology*.

[59] Sahid M. N. A., Kiyoi T. (2020). Mast cell activation markers for *in vitro* study. *Journal of Immunoassay and Immunochemistry*.

[60] Zhang Y., Li X., Lo C. K., Guo S. T. (2010). Characterization of the volatile substances and aroma components from traditional soypaste. *Molecules*.

[61] Chai W. M., Liu X., Hu Y. H. (2013). Antityrosinase and antimicrobial activities of furfuryl alcohol, furfural and furoic acid. *International Journal of Biological Macromolecules*.

[62] Yanagimoto K., Lee K. G., Ochi H., Shibamoto T. (2002). Antioxidative activity of heterocyclic compounds found in coffee volatiles produced by Maillard reaction. *Journal of Agricultural and Food Chemistry*.

[63] Slaughter J. C. (1999). The naturally occurring furanones: formation and function from pheromone to food. *Biological Reviews of the Cambridge Philosophical Society*.

[64] Miyake T., Shibamoto T. (1998). Inhibition of malonaldehyde and acetaldehyde formation from blood plasma oxidation by naturally occurring antioxidants. *Journal of Agricultural and Food Chemistry*.

[65] Weber V., Coudert P., Rubat C. (2002). Novel 4,5-diaryl-3-hydroxy-2(5H)-furanones as anti-oxidants and antiinflammatory agents. *Bioorganic & Medicinal Chemistry*.

[66] Tabaraki R., Yosefi Z., Ali H., Gharneh A. (2012). Chemical composition and antioxidant properties of *Malva sylvestris* L. *Agricultural Science Research Journal*.

