# Supplementary material for: Optimizing the Fermentation Conditions of Cudrania tricuspidata Fruit Using Bacillus amyloliquefaciens for Anti-Inflammatory Activity and GC-MS-Based Volatile Component Characteristics
Source: Evid Based Complement Alternat Med. 2023 Oct 18;2023:5042416. doi: 10.1155/2023/5042416 (PMC10599871; doi:10.1155/2023/5042416)
Supplement: Supplementary Materials — Figure S1 shows the plot of actual vs. predicted response for nitric oxide inhibition rate. Figure S2 and Figure S3 show the total ion chromatograph of nonfermented and fermented C. tricuspidata, respectively. [file 5042416.f1.docx]

Supplementary data

| 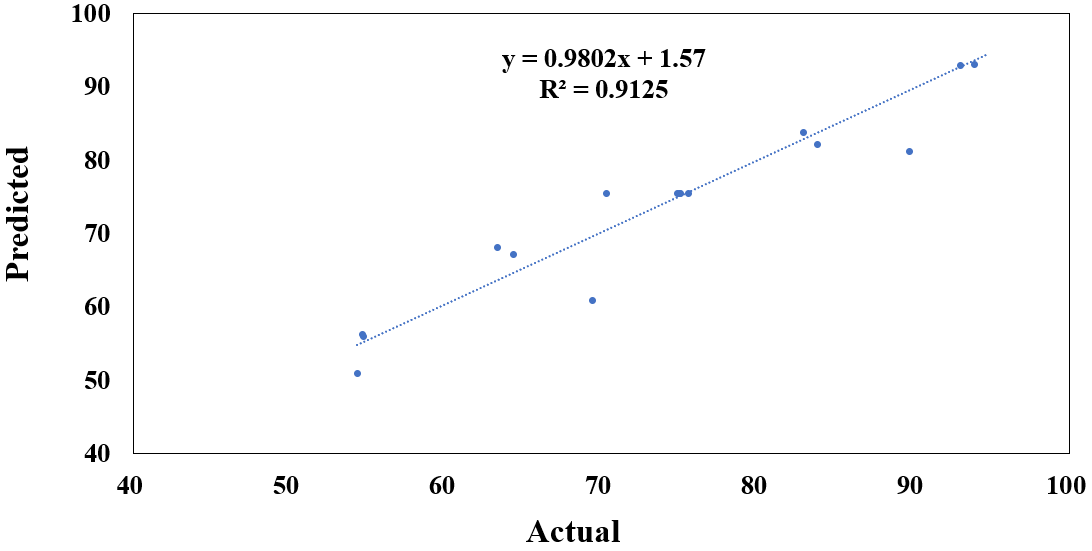 |
| --- |
| Fig. S1. Plot of actual vs. predicted response for nitric oxide inhibition rate. |

| 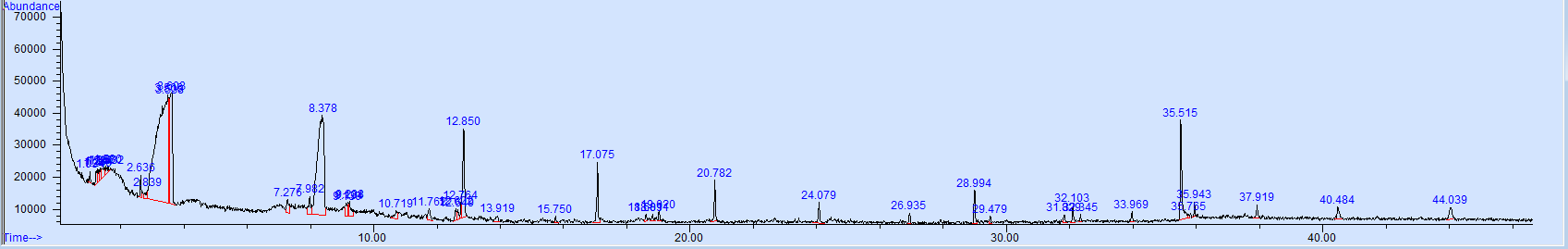 |
| --- |
| Fig. S2. Total ion chromatograph of non-fermented *C. tricuspidate* |

| 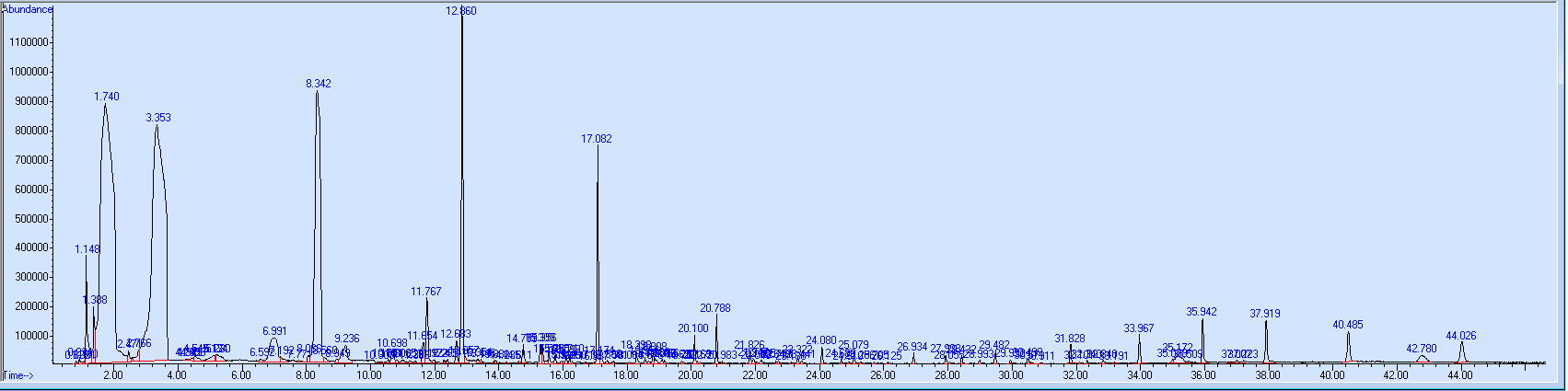 |
| --- |
| Fig. S3. Total ion chromatograph of fermented *C. tricuspidate* |
